# Addressing spatial bias in intracranial EEG functional connectivity analyses for epilepsy surgical planning

**DOI:** 10.1088/1741-2552/ac90ed

**Published:** 2022-09-23

**Authors:** Erin C Conrad, John M Bernabei, Nishant Sinha, Nina J Ghosn, Joel M Stein, Russell T Shinohara, Brian Litt

**Affiliations:** 1 Department of Neurology, University of Pennsylvania, Philadelphia, PA, United States of America; 2 Center for Neuroengineering and Therapeutics, University of Pennsylvania, Philadelphia, PA, United States of America; 3 Department of Bioengineering, University of Pennsylvania, Philadelphia, PA, United States of America; 4 Department of Radiology, University of Pennsylvania, Philadelphia, PA, United States of America; 5 Department of Biostatistics, Epidemiology and Informatics, University of Pennsylvania, Philadelphia, PA, United States of America; 6 Penn Statistics in Imaging and Visualization Center, University of Pennsylvania, Philadelphia, PA, United States of America; 7 Center for Biomedical Image Computing and Analytics, University of Pennsylvania, Philadelphia, PA, United States of America

**Keywords:** intracranial EEG, network analysis, drug-resistant epilepsy, epileptiform discharges, functional connectivity, spatial bias

## Abstract

*Objective.* To determine the effect of epilepsy on intracranial electroencephalography (EEG) functional connectivity, and the ability of functional connectivity to localize the seizure onset zone (SOZ), controlling for spatial biases. *Approach.* We analyzed intracranial EEG data from patients with drug-resistant epilepsy admitted for pre-surgical planning. We calculated intracranial EEG functional networks and determined whether changes in functional connectivity lateralized the SOZ using a spatial subsampling method to control for spatial bias. We developed a ‘spatial null model’ to localize the SOZ electrode using only spatial sampling information, ignoring EEG data. We compared the performance of this spatial null model against models incorporating EEG functional connectivity and interictal spike rates. *Main results.* About 110 patients were included in the study, although the number of patients differed across analyses. Controlling for spatial sampling, the average connectivity was lower in the SOZ region relative to the same anatomic region in the contralateral hemisphere. A model using intra-hemispheric connectivity accurately lateralized the SOZ (average accuracy 75.5%). A spatial null model incorporating spatial sampling information alone achieved moderate accuracy in classifying SOZ electrodes (mean AUC = 0.70, 95% CI 0.63–0.77). A model incorporating intracranial EEG functional connectivity and spike rate data further outperformed this spatial null model (AUC 0.78, *p* = 0.002 compared to spatial null model). However, a model incorporating functional connectivity without spike rate data did not significantly outperform the null model (AUC 0.72, *p* = 0.38). *Significance.* Intracranial EEG functional connectivity is reduced in the SOZ region, and interictal data predict SOZ electrode localization and laterality, however a predictive model incorporating functional connectivity without interictal spike rates did not significantly outperform a spatial null model. We propose constructing a spatial null model to provide an estimate of the pre-implant hypothesis of the SOZ, and to serve as a benchmark for further machine learning algorithms in order to avoid overestimating model performance because of electrode sampling alone.

## 1. Introduction

Emerging evidence suggests that aberrant connectivity may localize seizure generators in patients with drug-resistant epilepsy [1–9], and network analyses could be useful in identifying such aberrant connectivity profiles from intracranial electroencephalography (EEG) [7, 8, 10]. Intracranial EEG network analysis provides complementary advantages over network analysis applied to structural imaging, functional imaging, or scalp EEG, such as identifying functional state-dependent and frequency-dependent connectivity, excellent temporal and spatial resolution, and good signal-to-noise ratio [11–14]. Thus, intracranial EEG connectivity may inform localizing the *seizure onset zone (SOZ)*, the current clinical gold standard in estimating seizure generators.

**Figure 1.**
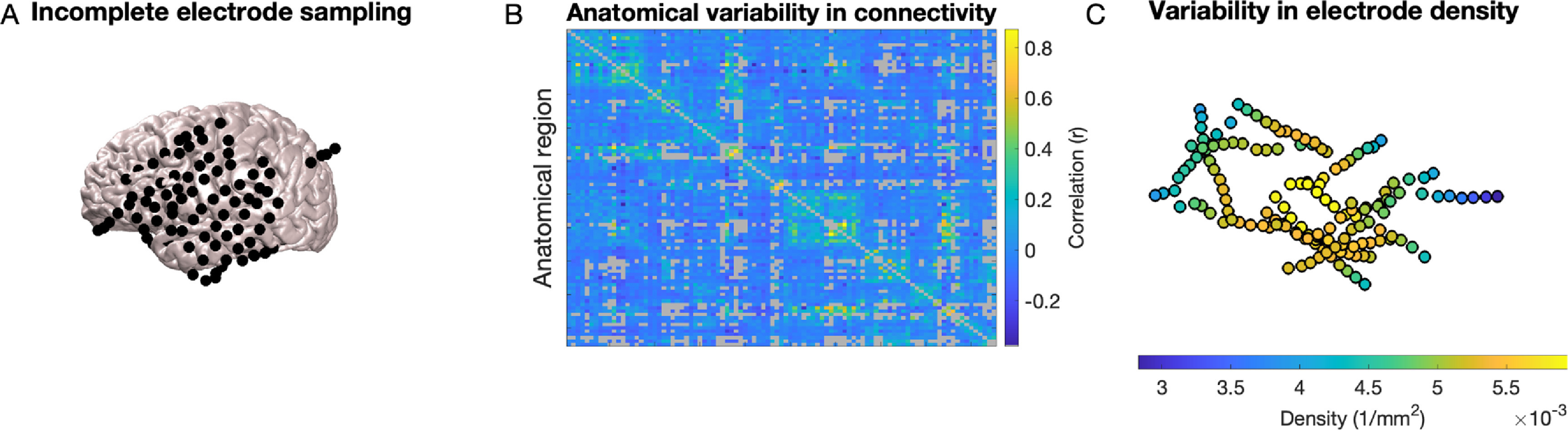
Spatial confounders bias intracranial EEG network analyses. (A) Electrodes are placed according to clinical needs, and thus electrode coverage is incomplete and variable across patients. (B) There is anatomic variability in connectivity, and it is unclear how much variability results from normal anatomical differences as opposed to epileptic changes. (C) Clinicians preferentially place electrodes around the suspected seizure generators, and thus, in the case of stereo-EEG, there is usually denser electrode coverage around suspected seizure generators. This may bias connectivity measurements, resulting in higher connectivity surrounding seizure generators by virtue of lower inter-electrode distance.

However, network analysis of intracranial EEG is subject to important potential spatial confounders (figure 1): First, it is challenging to determine whether inter-regional variability in connectivity reflects pathological epilepsy-related changes or normal anatomical variability [15–17]. Second, spatial sampling is limited by clinical necessity to where clinicians choose to implant electrodes [10, 11, 18, 19]. If clinicians sample the predicted location of seizure generators more densely, then we might expect higher connectivity measurements in the SOZ due to dense spatial sampling alone [10, 14, 17, 20]. These confounders currently limit our understanding of intracranial EEG connectivity and its utility in surgical planning. We need approaches to address these confounders both to better understand how epilepsy affects functional connectivity and to avoid biases in algorithms that use functional connectivity to localize the SOZ.

In this study, we analyzed intracranial EEG functional connectivity in patients with drug-resistant epilepsy undergoing intracranial presurgical evaluation. We employed multiple approaches to control for the biases highlighted above, investigating three main questions: (a) What is the effect of epilepsy on intracranial EEG connectivity? (b) How accurately can intracranial EEG functional connectivity localize and lateralize the SOZ? And (c) How can studies of intracranial EEG connectivity control for spatial bias? We found that (a) SOZ electrodes and SOZ regions demonstrate reduced connectivity, (b) reduction in connectivity predicts SOZ laterality, and (c) a spatial null model controlling for the choice of electrode placement estimates the clinicians’ pre-implant hypothesis of seizure generators, and this null model can serve as a benchmark to assess the performance of future predictive models using intracranial EEG.

## 2. Methods

### 2.1. Patient selection, determining SOZ electrodes and epilepsy lateralization, and electrode localization

This study was approved by the Hospital of the University of Pennsylvania (HUP) Institutional Review Board. Written informed consent was obtained from each participant.

We performed a retrospective analysis of all sequentially participating patients with drug-resistant epilepsy who underwent intracranial EEG recording as part of presurgical planning at HUP from January 2015–October 2021. Electrode targets were chosen according to the clinical pre-implant hypothesis of the seizure generators. In cases of suspected unilateral seizure generators, electrodes were sometimes implanted in the contralateral hemisphere if there was conflicting pre-implantation data (e.g. if there were frequent contralateral interictal spikes on scalp EEG or if the PET lesion was contralateral to other clinical data).

Seizures, SOZ electrodes, and the seizure onset lateralization (left, right, or bilateral) were identified by a board-certified epileptologist as part of the clinical evaluation, and confirmed at an epilepsy surgery case conference. Patients without seizures during the recording period were defined to have no SOZ electrodes and no seizure onset lateralization for the purpose of this study.

Pre-implant T1-weighted Magnetization-Prepared Rapid Acquisition Gradient Echo (MPRAGE) magnetic resonance imaging (MRI), post-implant T1-weighted MPRAGE MRI, and post-implant computed tomography images were acquired to localize electrodes for both the original and revised implantations. In-house software [21] was used to localize electrodes after registration of pre-implant and post-implant neuroimaging data. All electrode coordinates and labels were saved and matched with electrocorticography/stereo-EEG electrode names on IEEG.org. All electrode localizations were verified by a board-certified neuroradiologist.

### 2.2. Intracranial EEG recording and processing and spike detection

The supplemental methods section fully describes our approach to EEG recording, pre-processing, and spike detection. Briefly, we recorded intracranial EEG using a combination of grid, strip, and depth electrodes (including stereo-EEG), according to clinical needs. We performed automated rejection of intracranial EEG time segments detected to have heavy artifact, then applied a common average reference to intracranial EEG signals, and subsequently notch-filtered (removing 60 Hz noise) and bandpass-filtered (1–70 Hz) the resulting signals. We performed automated spike detection using a modified version of a previously published algorithm [22]. We validated automated spike detections using visual analysis of a random subset of detections.

### 2.3. Functional network calculation

We calculated functional networks in each one-minute segment. We performed our primary analysis using Pearson correlation as the connectivity metric. We divided segments into two-second non-overlapping windows. We defined the functional network for each two-second window to be an N_electrodes_ × N_electrodes_ matrix, where N_electrodes_ is the number of electrodes, and each *i, j* element of the matrix represents the (signed) Pearson correlation coefficient between intracranial EEG signals on electrodes *i* and *j*. We averaged the functional network across all two-second time windows, yielding one N_electrodes_ × N_electrodes_ functional network for each one-minute segment. The choice of two-second window and subsequent averaging over longer windows has been used in prior studies to analyze time-varying networks. [10, 23–26] We specifically chose to study one-minute segments in order to perform further analyses of time-varying functional connectivity that were outside the scope of this project, and thus subsequent analyses in this project averaged networks across all time periods to obtain a single time-averaged network for each patient. As a secondary analysis to test frequency-specific relationships, we also calculated functional networks using coherence as our connectivity metric. Using five canonical frequency bands (delta: 0.5–4 Hz, theta: 4–8 Hz, alpha: 8–12 Hz, beta: 12–30 Hz, and gamma: 30–80 Hz), we defined the *i, j* element of the adjacency matrix for each frequency band to be the magnitude squared coherence (calculated using Matlab’s mscohere, with a 2 s window and an overlap of 1 s) for that frequency band.

### 2.4. Atlas parcellation

As part of a method to control for spatial density of electrodes and to identify bilateral regions, we parcellated brain regions into a standard brain atlas. We performed our primary analysis using the Brainnetome atlas [27] and performed a secondary analysis using the automated anatomical labeling (AAL) atlas [28] in order to test the sensitivity of our results to atlas choice. For each electrode, we identified the atlas region nearest the electrode using montreal neurological institute (MNI) coordinates. We re-calculated functional networks and spike rates in this atlas space. For the univariate measure of spike rates, we defined the spike rate of a particular atlas region to be the mean spike rate of all electrodes belonging to that region. For bivariate measures (functional networks), we defined the network edge connecting atlas region *i* to atlas region *j* to be the average connectivity between the electrodes in region *i* and the electrodes in region *j*. Atlas network edges falling along the diagonal of the atlas adjacency matrix (representing the connectivity between electrodes in region *i* with other electrodes in region *i*) were defined to be unknown values. We defined SOZ regions to be those regions containing any SOZ electrodes.

### 2.5. Measuring SOZ inter-regional connectivity using symmetric coverage atlases

We next analyzed the connectivity in the SOZ region compared to the contralateral hemisphere. We constructed a *symmetric coverage atlas* to control for spatial sampling bias. To construct this atlas, for each patient, we identified those atlas regions with bilateral electrode coverage (electrodes in that atlas region on both the left and right hemispheres). We removed from analysis any atlas regions with only unilateral electrode coverage. This resulted in an artificially-constructed symmetric electrode coverage atlas that allowed us to compare connectivity in one hemisphere relative to the opposite side.

We first identified SOZ regions for each patient and compared the average connectivity of those regions to those in the contralateral hemisphere, treating each patient as an independent observation. In some cases, patients had multifocal or even bilateral SOZ regions (see table 1). In these cases, we included all SOZ regions, noting that this combined data from both hemispheres.

**Table 1.** Clinical and recording information.

Total: *N*	110
Female: *N* (%)	60 (54.5%)
Age at onset in years: median (range)	16.0 (0.0–59.0)
Age at implant in years: median (range)	36.5 (16.0–69.0)
SOZ laterality	
Left: *N* (%)	48 (43.6%)
Right: *N* (%)	33 (30.0%)
Bilateral: *N* (%)	25 (22.7%)
SOZ localization	
Mesial temporal: *N* (%)	35 (31.8%)
Temporal neocortical: *N* (%)	13 (11.8%)
Other cortex: *N* (%)	25 (22.7%)
Number of electrodes: median (range)	138.0 (50.0–281.0)
Implant type	
Grids/strips/depths: *N* (%)	15 (13.6%)
Stereo-EEG: *N* (%)	95 (86.4%)
Intracranial recording duration in days: median (range)	9.8 (2.0–27.7)

We next analyzed only patients with unilateral SOZs and compared the average intra-hemispheric connectivity on the side of the SOZ to that of the contralateral side. Finally, we restricted analysis to patients whose SOZ spanned multiple atlas regions and who had electrode coverage of these regions and the equivalent regions in the contralateral hemisphere. We then compared the intra-SOZ connectivity (the connectivity between SOZ regions) to that of the contralateral side. We used Wilcoxon signed-rank tests for these comparisons, reporting the positive-rank sum *T^+^* as the test statistic. This method studies only inter-regional connectivity, ignoring connectivity between electrodes within the same atlas region.

Importantly, we might expect a remaining bias toward a higher number of electrodes in the region of the SOZ compared to the same region in the contralateral hemisphere. We anticipated that this might lead to a lower variance in the average connectivity involving the SOZ region (because more individual electrodes’ connections contribute to the average inter-regional connectivity), but not a bias in the average connectivity. To test this, we repeated the above analyses, this time randomly subsampling electrodes in each region when calculating regional connectivity such that the number of electrodes in a given region equaled the number of electrodes in its corresponding contralateral region. We recalculated the average connectivity in the SOZ compared to the contralateral region (separately for each of the three types of SOZ connectivity defined above). We performed this resampling 100 times and measured the average across resamples, again performing Wilcoxon signed-rank tests on these subsample-averaged values. We expected the resulting statistics to be similar to those in the above non-subsampled analysis.

## 3. Lateralizing the SOZ using functional connectivity and spikes

We next developed a model to predict SOZ laterality using functional connectivity and spikes. We restricted our analysis to patients with unilateral SOZ and at least two symmetrically-implanted atlas regions in each hemisphere, which allowed us to calculate intra-hemispheric connectivity. Using the symmetric coverage atlas (restricting regions to symmetric coverage), we measured the average left hemispheric connectivity and the average right hemispheric connectivity, as well as average left and right hemispheric spike rates. We constructed a logistic regression classifier, where each patient was a separate observation, and the response variable was a binary indicator of left-sided or right-sided SOZ. We used three separate models:

• Model 1 (*Connectivity*) Predictors: left intra-hemispheric average connectivity, right intra-hemispheric average connectivity.
• Model 2 (*Spike rates*) Predictors: left hemispheric average spike rates, right hemispheric average spike rates.
• Model 3 (*Connectivity + spike rates*) Predictors: left intra-hemispheric average connectivity, right intra-hemispheric average connectivity, left hemispheric average spike rates, right hemispheric average spike rates.

We used a leave-one-patient-out cross validation approach, leaving one patient as testing data and training the model on the remaining patients. We defined a classifier threshold of 0.5 for predicting left vs right-sided epilepsy laterality, and constructed a confusion matrix comparing the resulting individual test patient predictions against the true SOZ lateralizations.

### 3.1. Construction of a spatial null model to predict the SOZ

Machine learning predictions of the SOZ using intracranial EEG functional connectivity are limited by spatial bias. To quantify this bias, we constructed a null model that predicted whether an electrode belonged to the SOZ based entirely on electrode sampling, before consideration of intracranial EEG data. This model is based on the assumption that the pre-implantation hypothesis of seizure generators informs implantation strategy. We incorporated (a) anatomical information, given the expectation that certain anatomical regions would more likely belong to the SOZ, and (b) spatial density, hypothesizing that electrodes with a greater number of nearby electrodes would more likely belong to the SOZ (implying that clinicians sample the predicted SOZ more densely).

To model anatomical location, we assigned the atlas parcellation of each electrode into one of three regions: mesial temporal, temporal neocortical, and other cortex (figure 4(A)). Electrodes not belonging to any of these regions (e.g. white matter contacts and those outside the brain) were excluded from the model. We chose this coarse division for two reasons: (a) utilizing a large number of anatomical divisions leads to overfitting, and (b) the regions chosen are broad anatomical localizations that clinicians often consider when choosing electrode sampling.

To model spatial density, we calculated the Kernel density at each electrode resulting from the contribution of all other electrodes (figure 4(B)). The Kernel density (density) at each electrode *i* was defined as [29]:

\begin{equation*}{\text{Densit}}{{\text{y}}_i} = { }\frac{1}{{{\text{radiu}}{{\text{s}}^2}}}\mathop {\sum }\limits_{j = 1}^N \left[ {\frac{3}{\pi }{{\left( {1 - {{\left( {\frac{{{\text{dis}}{{\text{t}}_{\left( {i,j} \right)}}}}{{{\text{radius}}}}} \right)}^2}} \right)}^2}} \right].\end{equation*}

Where *N* is the number of other electrodes, dist*_(i,j)_* is the Euclidean distance between the current electrode *i* and electrode *j*, and radius is the search radius. If dist*_(i,j)_* > radius, then the density at electrode *i* from electrode *j* is defined to be 0 (only electrodes closer to electrode *i* than the search radius are included). We selected a patient-specific search radius defined as the distance between the two electrode contacts that were furthest away from each other, thus ensuring that every other electrode would be included in a given electrode’s density calculation.

An electrode with a higher Kernel density has more electrodes nearby (figure 4(C) shows the Kernel densities of the electrodes of a single patient as an example). We normalized the densities across electrodes within each patient by subtracting the mean across electrodes and dividing by the standard deviation.

### 3.2. Comparing performance of spatial null model to models incorporating intracranial EEG data

We compared the spatial null model to models incorporating intracranial EEG functional connectivity and spike rate data. For functional connectivity, we measured the average Pearson correlation over time for each electrode to the other electrodes in the network. For spike rates, we measured the average spike rate over time for each electrode. We normalized both the connectivity and spike rates within each patient by subtracting by the mean across electrodes and dividing by the standard deviation across electrodes. We constructed four models, with the following different sets of predictors:

• Model 1 (*Spatial null model*) predictors: anatomical location (mesial temporal, temporal neocortical, or other cortex) and spatial density (as defined in the preceding subsection).
• Model 2 (*Spatial null model + connectivity*) predictors: anatomical location, spatial density, and average functional connectivity.
• Model 3 (*Spatial null model + spikes*) predictors: anatomical location, spatial density, and average spike rate.
• Model 4 (*Spatial model + connectivity + spikes*): anatomical location, spatial density, average, functional connectivity, and average spike rate.

To train the models, we randomly selected ⅔ of the patients to use as training data. We treated each electrode of each patient as a separate observation. We trained a logistic regression classifier (Matlab’s fitglm, Binomial distribution with logit link function). The response variable was a binary variable representing whether the electrode was in the SOZ or not. The predictors were those features noted above. We tested the model on the remaining ⅓ of patients, comparing the model’s probabilities that each electrode belongs to the SOZ against the true classification of SOZ or not using a receiver operator characteristic (ROC) curve. We performed 1000 random splits of training and testing data to obtain model statistics. We calculated each model’s ROC area under the curve (AUC) as an estimate of overall performance. To compare each model’s accuracy, for each pair of models we measured the difference between the two models’ AUCs for each random training/testing split. We defined the two-tailed *p*-value testing whether one model’s AUC was higher than another’s to be:


\begin{equation*}p = 2 \times \frac{{{\text{min}}\left[ {N\left( {{\text{AU}}{{\text{C}}_{{\text{diff}}}} \leqslant 0} \right),{ }N\left( {{\text{AU}}{{\text{C}}_{{\text{diff}}}} \geqslant 0} \right)} \right] + 1}}{{{N_{{\text{total}}}} + 1}}\end{equation*}

Where *N*_total_ is the total number of training/testing splits, *N*(AUC_diff_ ⩽0) is the number of differences in the two models’ AUCs that are less than or equal to zero, and *N*(AUC_diff_ ⩾ 0) is the number of differences in the two models’ AUCs that are greater than or equal to zero. Note that with this method, the lowest possible *p-*value would occur in the case in which all AUC differences are less than zero (or all are greater than zero), in which case the *p*-value would be *p* = 2 × 1/1001 = 0.002.

We anticipated that model performance might be better for patients with stereo-EEG implantations compared to those with grid/strip/depth implantations. This is because electrode spacing is uniform in grids and strips, and so spatial density in these implantations does not reflect clinician suspicion of seizure generators. Therefore, the spatial null model (which is a component of all subsequent models) would likely underperform in grid/strip/depth implantations. To test this, we separated patients into those with stereo-EEG implantations and those with grid/strip/depth implantations. We repeated the machine learning approach described above (with 1000 random splits of ⅔ of patients as training data and ⅓ of patients as testing data) separately for the two implantation strategies. We used the same approach above to calculate a two-tailed *p-*value determining whether the difference in model AUCs between each pair of models was less than or greater than zero.

Finally, we used the full model (incorporating null information, functional connectivity, and spikes) to determine the association between an electrode’s functional connectivity and its likelihood of being in the SOZ controlling for these other variables. For this analysis, we studied all patients and used a logistic regression mixed effects model (Matlab’s fitglme, Binomial distribution with logit link function) with the patient identifier as a random effect. We used bootstrapping (selecting from patients randomly with replacement, *N* = 1000) to generate statistics for the model coefficients.

### 3.3. Statistical analysis

All analysis was performed in Matlab R2021a (Mathworks, Natick, MA). Statistical analyses are described in the specific sections above.

### 3.4. Code availability

All code used to perform the analysis, along with an intermediate dataset needed to perform statistical analyses, is available on Github: https://github.com/erinconrad/FC_toolbox/tree/main/analyses/outcome_prediction/plots/paper_plots.

## 4. Results

We included all patients who had available electrode localizations (110 patients), although the number of patients analyzed varied by analysis, as noted in the results of individual analyses. Patients were heterogeneous by age, sex, SOZ localization and lateralization, and implant strategy (table 1).

### 4.1. Connectivity with symmetric coverage constraint

**Figure 2.**
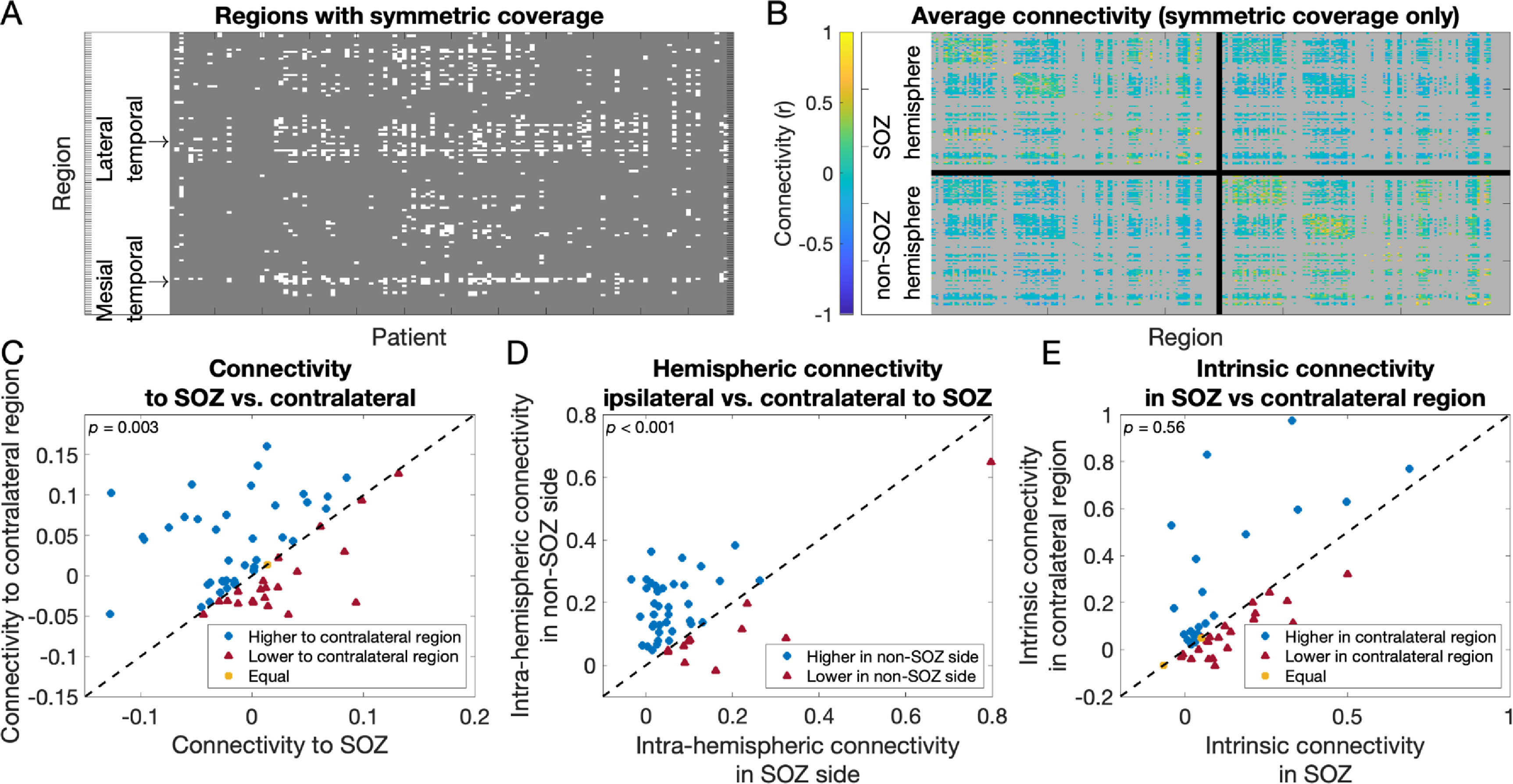
Intracranial EEG connectivity in anatomically symmetric brain networks. (A) Anatomic regions with symmetric electrode coverage. The *x*-axis indicates which patient and the *y*-axis indicates which anatomic region in the Brainnetome atlas. White regions are those with symmetric coverage (for which there are electrodes in the corresponding region in both hemispheres) for a given patient. Gray regions have either asymmetric coverage (electrodes in only one hemisphere) or no coverage. Groups of regions corresponding to the mesial temporal and lateral temporal cortex, which are commonly implanted symmetrically, are shown. (B) The average connectivity in a symmetric coverage network, across all patients. For each patient, we constructed an artificial symmetric coverage network, defining all network edges involving a region without symmetric electrode coverage to be unknown. The plot shows the average across all patients of their symmetric coverage networks. Both the *x*- and *y*- axes indicate the Brainnetome atlas region, now ordered such that the SOZ hemisphere is on the top and the non-SOZ hemisphere is on the bottom (patients without clear epilepsy laterality were excluded from this plot). The intra-hemispheric connectivity in the SOZ hemisphere (the edges in the top left quadrant) visually appeared lower (less yellow) than that of the non-SOZ hemisphere (bottom right quadrant). (C) Connectivity to the SOZ region versus the contralateral region. Each marker is one patient. Markers above the diagonal (blue circles) are patients for whom the average connectivity to the region contralateral to the SOZ is higher than that to the SOZ region. Markers below the diagonal (red triangles) are patients for whom the average SOZ regional connectivity is higher. Connectivity to the SOZ region tended to be lower than to the contralateral region. (D) The average intra-hemispheric connectivity (the connectivity between regions in a single hemisphere) was lower in the hemisphere of the SOZ compared to the contralateral hemisphere. (E) There was no difference in the intrinsic connectivity in the SOZ (the average connectivity between SOZ regions and other SOZ regions) and the contralateral regions.

To control for spatial sampling bias, we first compared connectivity in the SOZ against that in the contralateral region while restricting analysis to regions with symmetric electrode coverage. Across patients, there were on average 7.8 (range 0–28) anatomical regions (15.7 when separately counting left and right) with bilateral electrode coverage. 82 of 110 patients had regions with bilateral electrode coverage (figure 2(A)). Visually, in a network averaged across patients, network edges in the hemisphere opposite the SOZ region were stronger than those in the hemisphere of the SOZ region (figure 2(B), brighter colors indicate higher connectivity).

We compared the average connectivity to the SOZ region with that to the region contralateral to the SOZ. We included patients who had electrode coverage of both the SOZ region and the contralateral region in this analysis (62 patients). The average connectivity to the SOZ region (median 4.4 × 10^−4^) was lower than that to the region contralateral to the SOZ (median 9.1 × 10^−3^) (Wilcoxon signed-rank test: *T^+^* = 513.0, *p* = 0.003) (figure 2(C)).

We next compared the intra-hemispheric connectivity between the side of the SOZ and the contralateral hemisphere. For this analysis we included patients with unilateral epilepsy and at least two symmetrically-implanted atlas regions in each hemisphere, which allowed us to calculate intra-hemispheric connectivity (49 patients). The average intra-hemispheric connectivity on the side of the SOZ (median 5.2 × 10^−2^) was also lower than that in the contralateral hemisphere (median 1.4 × 10^−1^) (Wilcoxon signed-rank test: *T^+^* = 199.0, *p* < 0.001) (figure 2(D)). This suggests that the change in connectivity in epilepsy is broad and affects the entire hemisphere.

We next compared the intrinsic connectivity within the SOZ regions and that of the contralateral region. For this analysis we included patients whose SOZ spanned at least two atlas regions, and who had electrode coverage of these regions and of the contralateral regions (45 patients). The intrinsic connectivity within the SOZ (between one SOZ region and other SOZ regions) (median 6.7 × 10^−2^) was no different from the intrinsic connectivity within the same regions in the contralateral hemisphere (median 7.6 × 10^−2^) (Wilcoxon signed-rank test: *T^+^* = 425.0, *p* = 0.56) (figure 2(E)). This suggests no focal within-SOZ change in connectivity within the limits of this analysis. Results were similar when using the AAL rather than the Brainnetome atlas for parcellating brain regions (supplemental results; figure S1). Results were also similar in our supplemental analysis in which we subsampled electrodes to achieve equivalent numbers of electrodes contributing to a given region and its contralateral region (supplemental results; figure S2). Results when studying coherence rather than Pearson correlation networks were more heterogeneous, seen for different frequency bands in the two different atlases (supplemental results; figure S3).

### 4.2. Epilepsy lateralization

**Figure 3.**
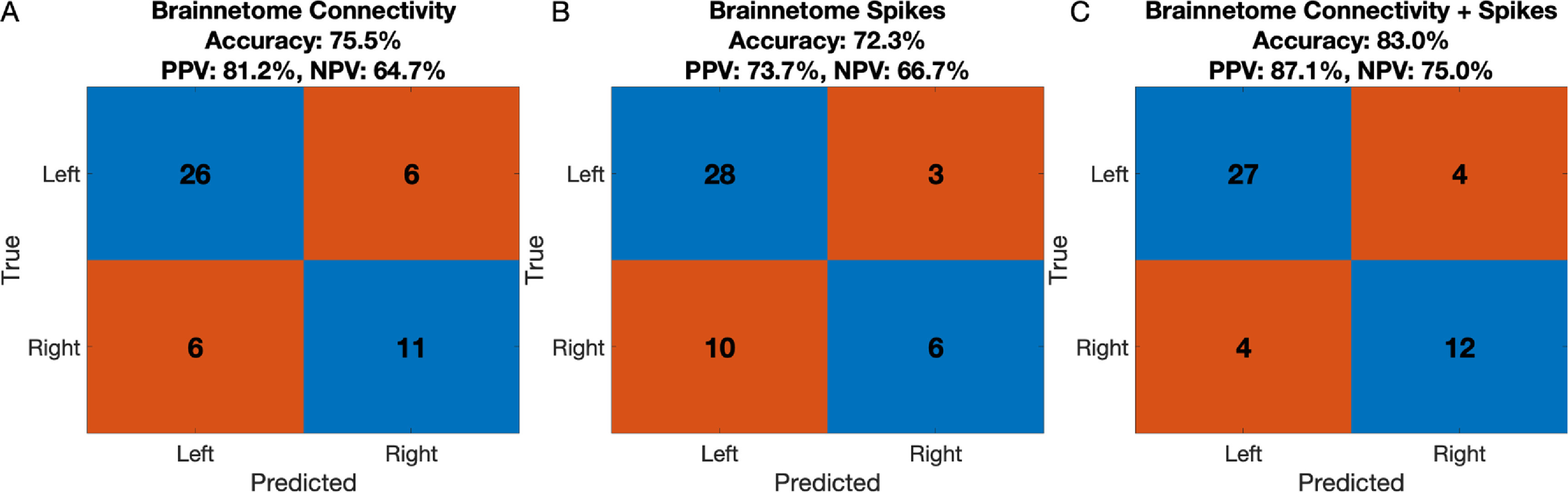
Intra-hemispheric connectivity lateralizes the SOZ. Each plot is a confusion matrix showing the accuracy of a model predicting the laterality of the SOZ in a held-out test patient, summed across all patients, using the Brainnetome atlas for regional parcellation. (A) shows the confusion matrix for a model incorporating intra-hemispheric connectivity, (B) shows the confusion matrix for a model incorporating spike rates, and (C) shows the confusion matrix for a model incorporating both functional connectivity and spike rates. The *y*-axis indicates the clinical designation of the SOZ lateralization (taken as the gold standard). The *x*-axis indicates the predicted laterality. Corresponding accuracies, positive predictive values (PPV), and negative predictive values (NPV) are shown.

We asked how well intra-hemispheric functional connectivity could lateralize epilepsy. We restricted analysis to patients with unilateral SOZ and at least two symmetrically-implanted atlas regions in each hemisphere, which allowed us to calculate intra-hemispheric connectivity (49 patients). The average accuracy of SOZ laterality predictions in the test patients was 75.5% (positive predictive value (PPV) for detecting left-sided seizure onset: 81.2%, negative predictive value (NPV): 64.7%) (figure 3(A)). This model performed similarly to one using spike rates rather than connectivity (figure 3(B)). Using a model based on the AAL atlas rather than the Brainnetome atlas yielded similar results (figure S4).

### 4.3. Predicting the SOZ

**Figure 4.**
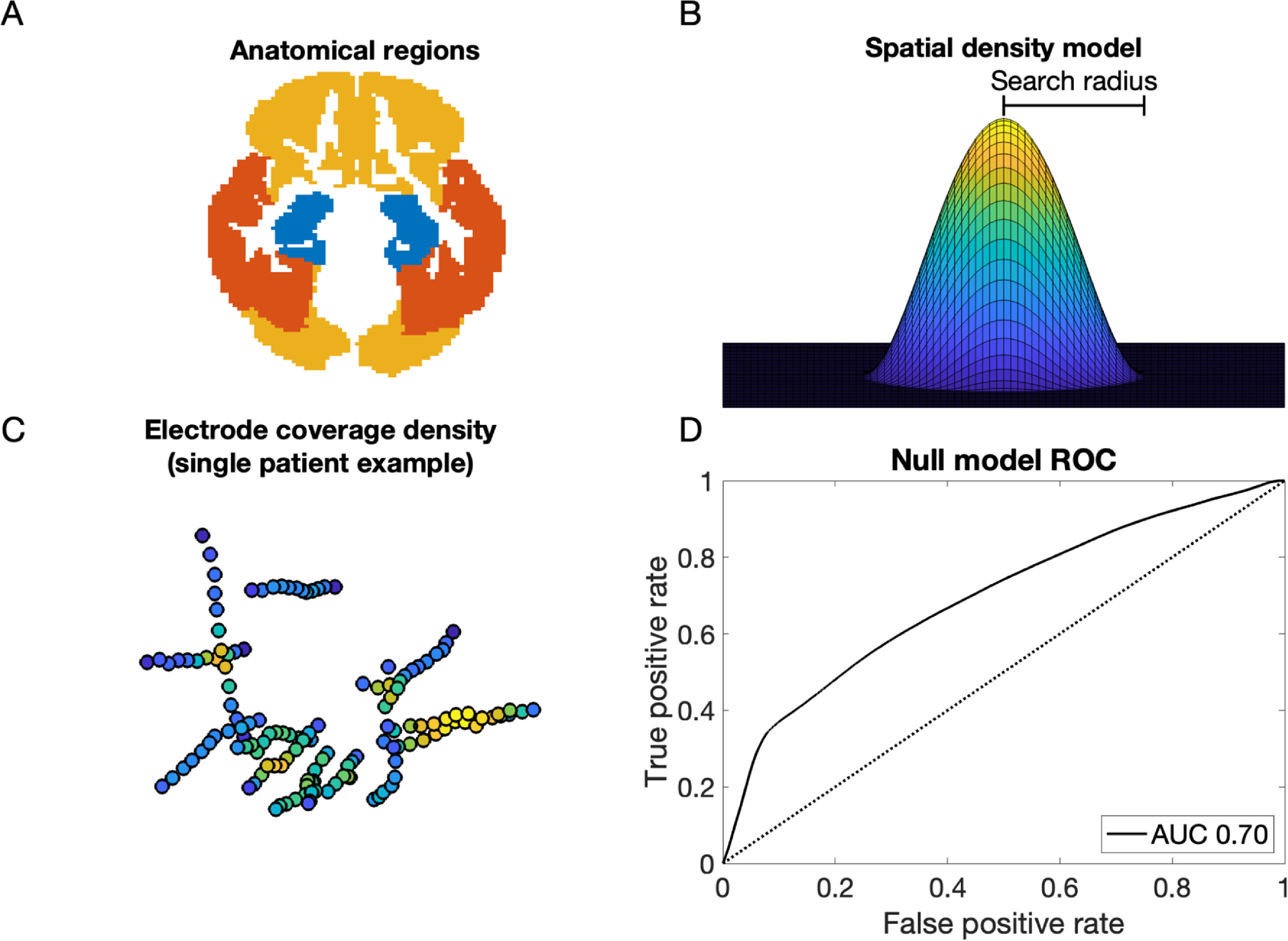
Constructing a spatial null model to localize the SOZ electrode. (A) To model the effect anatomy has on the likelihood of an electrode belonging to the SOZ, we performed coarse parcellation of the brain into three anatomic regions: the mesial temporal region (blue), temporal neocortical region (red), and other cortex (orange). Electrodes in white matter or outside the brain were discarded from this analysis. (B) To model the effect electrode sampling density has on the likelihood of an electrode belonging to the SOZ, we first calculated the Kernel density at each electrode, representing the density of neighboring electrodes. (C) The density at each electrode for a single patient example. More yellow colors indicate higher density, as seen at the electrodes with a higher number of closely neighboring electrodes. (D) We combined the anatomical information with the electrode coverage density and used these as predictors in a logistic regression classifier modeling the probability of an electrode belonging to the SOZ. The AUC of the resulting spatial null model was 0.70, indicating that a substantial amount of information about the SOZ electrode can be obtained from electrode coverage alone.

**Figure 5.**
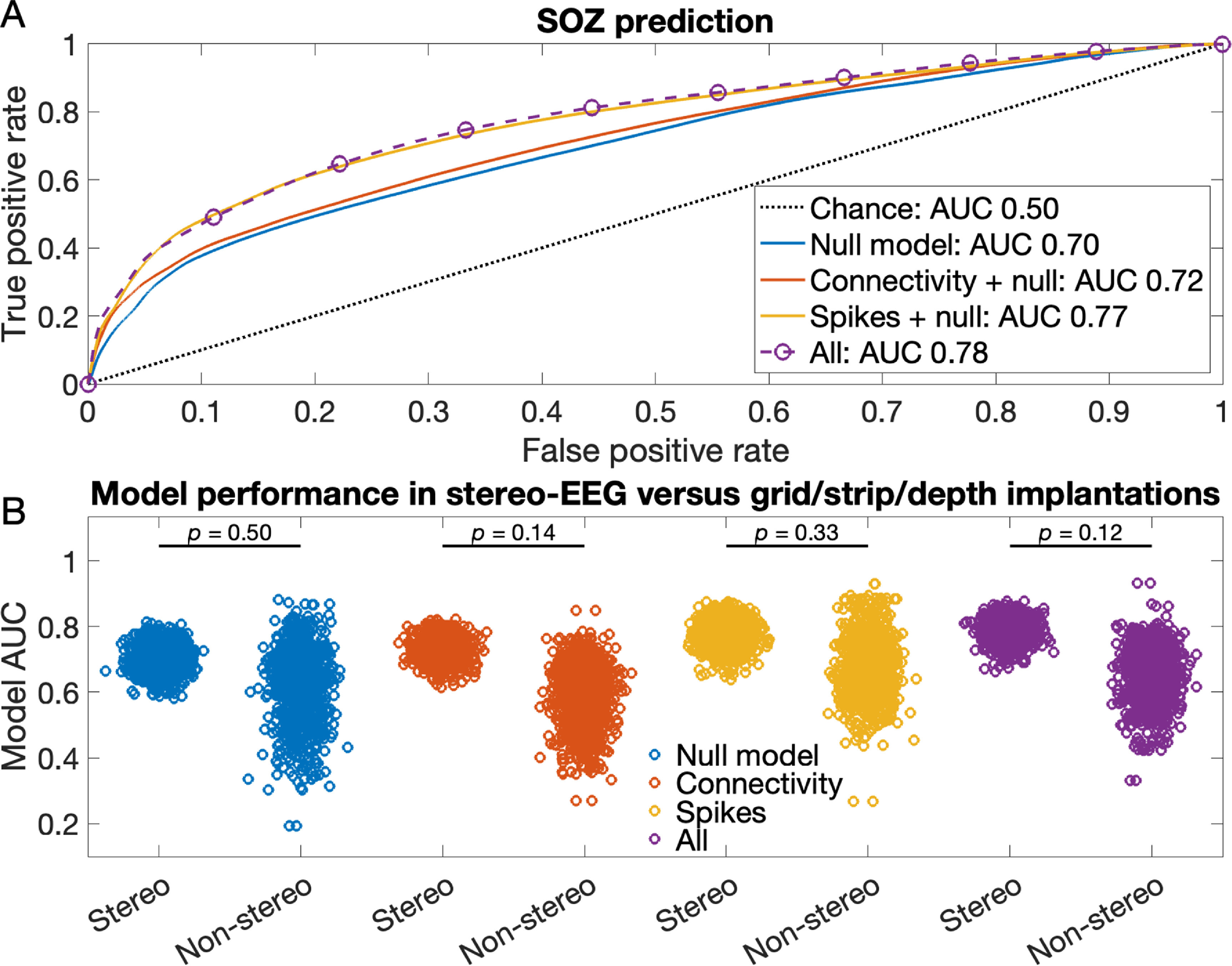
Performance of models incorporating interictal data at predicting the SOZ electrode. (A) ROC curves for multiple models predicting the SOZ electrode. All models were trained on ⅔ of the patients and tested on the remaining ⅓. About 1000 random training/testing splits were performed, and the resulting ROC curves and AUCs are averaged across all splits. Models include (a) the null model as described in figure 4, which contains only spatial sampling information and no intracranial EEG data, (b) a model including both the null model and connectivity data, (c) a model including the null model and spike data, and (c) a model including the null model, connectivity data, and spike data. The average AUC for the model containing connectivity data is only slightly (and non-significantly) higher than for the null model. A model containing both spike data and connectivity data outperforms the null model. (B) A comparison of model performances for patients with stereo-EEG versus grid/strip/depth (‘non-stereo’) implantation strategies. The same procedure was followed as in (A), however patients with stereo-EEG and non-stereo-EEG implants were separately analyzed. Each marker shows the AUC for the specified model for a given random training/testing split (out of the 1000 splits). Each model performed better in patients with stereo-EEG implantations than in patients with non-stereo-EEG implantations, though no difference was significant.

We next developed a classifier to predict whether an individual electrode contact belongs to the SOZ or not. To account for expected spatial bias in electrode sampling (clinicians preferentially place electrodes closer to the SOZ), we first constructed a null model, which provides an estimate of the accuracy of predicting the SOZ electrodes based entirely on spatial location (figure 4). This is an estimate of how accurately we can localize the SOZ electrodes before even considering intracranial EEG data. We compared the performance of the null model against a model including connectivity data, a model including spike rate data, and a model including both spikes and connectivity (figure 5(A)). This analysis included all patients with electrode localizations and accurate spike detections (94 patients). The model was trained on 2/3 of the patients, and tested on the remaining 1/3. About 1000 random splits of patients into testing and training data were performed to obtain model statistics. These results show that, first, even a null model based upon electrode placement, ignoring intracranial EEG data substantially outperforms a chance model (null model AUC = 0.70, 95% CI 0.63–0.77). It also shows incremental improvement in adding connectivity data (AUC = 0.72; albeit not as good as with adding spike data, AUC = 0.77), with still better performance when combining spike and connectivity data (AUC = 0.78; table 2 shows statistics of model comparisons). Notably, using a conservative two-sided bootstrap method to compare AUCs of different models, a model adding functional connectivity data did not significantly outperform the spatial null model alone (*p* = 0.38).

**Table 2.** Comparison of model performance for localizing the seizure onset zone (SOZ) electrodes. The first row shows the mean and 95% confidence interval (CI) area under the curve (AUC) corresponding to the receiver operator characteristic (ROC) curves for models trained on ⅔ of patients and tested on the remaining ⅓, across 1000 random training/testing splits. Other rows demonstrate comparisons between different models. Statistics shown are two-tailed *p*-values testing whether performance between the corresponding model pair is significantly different. These results indicate that a model incorporating functional connectivity does not significantly outperform a spatial null model. A model incorporating spikes, as well as a model incorporating spikes and functional connectivity, both outperform a spatial null model.

	Null model	Null + connectivity	Null + spikes	All
Model performance	Mean AUC 0.70	Mean AUC 0.72	Mean AUC 0.77	Mean AUC 0.78
	95% CI [0.63–0.77]	95% CI [0.66–0.79]	95% CI [0.70–0.84]	95% CI [0.72–0.84]
Null model	—	*p =* 0.38	*p =* 0.002	*p =* 0.002
Null + connectivity	—	—	*p =* 0.020	*p =* 0.002
Null + spikes	—	—	—	*p =* 0.56
All	—	—	—	—

We anticipated that the spatial null model and other models (all of which incorporate the spatial null model information) might perform better in patients with stereo-EEG than in patients with grid/strip/depth implantations because electrode spacing is uniform in grids and strips. To test this, we compared the performance of models trained and tested only on patients with stereo-EEG implantations (81 patients who also had accurate spike detections and electrode localizations) against those trained on patients with grid/strip/depth implantations (13 patients). The performance of each model was higher in the case of stereo-EEG implantations, although the distributions heavily overlapped, and no difference was significant (figure 5(B); table S2).

Finally, we used the estimate of the model coefficients to assess how an electrode’s connectivity is associated with the likelihood of being in the SOZ, controlling for spatial bias and spike rates. For this analysis we used a logistic mixed effects model trained on all patients. The patient identifier was a random effect. Holding covariates constant, the odds of an electrode being a SOZ electrode decreased by 33.1% (bootstrap 95% CI OR [0.57–0.79]) for each additional normalized connectivity unit (bootstrap *p* = 0.002). The odds ratio less than 1 implies that, controlling for spatial sampling bias and spike rates, a lower average connectivity increases the likelihood of an electrode being in the SOZ.

## 5. Discussion

In this study, we measured functional connectivity in patients with focal epilepsy, exploring multiple methods to control for spatial biases inherent in studying intracranial EEG networks resulting from the fact that clinicians place electrodes more densely around the suspected seizure generators. We report two primary findings: (a) Controlling for spatial sampling bias, the SOZ region has lower connectivity to the rest of the brain than contralateral control regions (figure 2), and this can predict SOZ laterality (figure 3). (b) A model predicting the SOZ electrodes incorporating only spatial sampling information achieves better-than-chance classification performance (mean AUC 0.70, 95% CI 0.63–0.77, figure 5), highlighting the importance of using a spatial null model to avoid overestimating model performance.

### 5.1. The SOZ region has reduced connectivity

Restricting analysis to regions with symmetric electrode coverage, the average connectivity to the SOZ region is lower than to the same region in the opposite hemisphere (figure 2(C)). We also found that the intra-hemispheric connectivity of the entire hemisphere on the same side of the SOZ region was reduced compared to the contralateral hemisphere (figure 2(D)). This suggests that epilepsy may be associated with a broad holo-hemispheric reduction in connectivity. By parcellating the brain and measuring inter-regional connectivity, this method ignores intra-regional connectivity, studying only longer range connections. Also, by performing hemisphere-to-hemisphere comparisons, we restrict analysis to patients with bilateral implantations, who might have different networks from those clinicians chose to implant unilaterally. Our model classifying individual electrodes as SOZ or non-SOZ similarly found that SOZ electrodes tend to have lower average functional connectivity controlling for spatial sampling and spike rates. This separate approach avoids both limitations noted above.

Prior studies of structural networks have also found reduced connectivity that localizes the SOZ [30–32]. The intracranial EEG network literature is less clear, with conflicting findings of both reduced and increased connectivity [7, 14]. Shah *et al* [7] found increased interictal functional connectivity in the local network comprising the resected region in epilepsy patients (used as a surrogate for the seizure generators). Here we found no difference in intrinsic connectivity between SOZ regions and the contralateral regions (figure 2(E)), although our analysis was limited in that it only included patients whose SOZs spanned multiple atlas regions. Studies in MEG and intracranial EEG suggest the coexistence of both a local *increase* in connectivity involving very focal seizure generators and a broad *decrease* in connectivity between the seizure generators and more distant regions [14, 33].

A limitation of the symmetric coverage method we used in our analysis is that it does not control for differences in the *intra*-regional electrode numbers. Our supplementary analysis suggests that these differences do not affect the average *inter*-regional connectivity (figure S2). However, we expect that potential biases in intra-regional electrode numbers will affect both the variance in inter-regional connectivity measurements and any *intra-*regional connectivity measurements, limiting applicability to potential future analyses.

### 5.2. Intra-hemispheric connectivity lateralizes epilepsy

The average intra-hemispheric connectivity in a network reduced to regions with symmetric electrode coverage correctly lateralized the SOZ 76% of the time, similar to spike rate data (figure 3). The moderate performance of this model suggests that neither spikes nor functional connectivity are likely to be sufficient to lateralize epilepsy. However, they may add useful ancillary data in cases in which ictal intracranial data is insufficient, for instance when the seizure onset pattern is unclear or the patient is seizure-free after prolonged recording.

### 5.3. Intracranial EEG spatial sampling (i.e. clinical judgment) predicts the SOZ electrodes

A model using only information about where electrodes were placed exceeded chance (AUC 0.70, 95% CI 0.63–0.77) at predicting SOZ electrodes. It is challenging to compare this null result to that of other published analyses because those of which we are aware all predict somewhat different things. Noting that limitation, to put our result into perspective, a study using intracranial EEG networks to identify the epileptogenic zone in patients with good surgical outcome reported an AUC of 0.92 [34], a study using intracranial EEG networks to separate resected SOZ from normal brain reported an AUC of 0.77 [15], and a study using intracranial EEG networks to predict good or bad surgical outcome reported an AUC of 0.70 [10]. The better-than-chance performance of our null model reflects that clinicians’ prior expectations about the location of seizure generators drive their choice of electrode placement. A similar model could serve as a benchmark against which to test future machine learning algorithms that use intracranial EEG data to localize seizure generators. To conclude that an intracranial EEG feature is a potentially useful biomarker for surgical planning, a model incorporating this feature should outperform a spatial null model. A model that does not outperform the spatial null model implies that the biomarker being studied does not provide more information than what clinicians knew before looking at the intracranial EEG data.

Importantly, future spatial null models that outperform the one presented here could likely be constructed. Another limitation of the spatial null model is that it itself is subject to bias: clinicians choose electrode placement and they also determine the SOZ electrodes. Future studies could compare a spatial null model against a model informed by intracranial EEG data in their ability to predict surgical outcome, rather than the clinician-defined SOZ electrodes, which would avoid this circularity. Finally, implantation strategies vary across institutions (for instance, an institution may conceivably place electrodes in similar locations across patients regardless of the pre-implantation seizure generator hypothesis), and therefore the external validity of our proposed spatial null model likely varies across institutions.

### 5.4. Incorporating interictal data improves prediction of the SOZ electrodes

A model incorporating functional connectivity and spike rates outperforms a spatial null model at localizing the SOZ electrodes, providing additional evidence that interictal data can help identify seizure generators [7, 10, 14, 34]. Importantly, a model including functional connectivity without spikes did *not* significantly outperform a spatial null model at localizing the SOZ electrodes. This negative result is somewhat limited by the conservative bootstrap analysis, but it suggests that, while reduced functional connectivity likely localizes the SOZ electrodes, its independent predictive effect is small in our study. We do *not* claim that functional connectivity more generally does not predict the SOZ electrodes. Given that the scope of this study was to examine and control for spatial biases in intracranial EEG network analysis rather than to optimize a SOZ predictor, we did not exhaustively examine other features (e.g. choice of connectivity measure, different temporal sampling) that may outperform the choices in this study [34], and so it is very likely that a network-based model could outperform a spatial null model. A recent study found that normalizing for spatial density improves predictions of surgical outcome in network analysis relative to a non-normalized model, which provides evidence that intracranial EEG functional networks independently localize seizure generators beyond just spatial sampling alone [10]. Regardless, the limited performance of our network model beyond the spatial null model underscores how using a spatial null model rather than a chance null model provides a more conservative and realistic estimate of the additive utility of functional connectivity.

## 6. Conclusions

Network analysis of interictal intracranial EEG is a promising approach to improve our understanding and treatment of epilepsy, but is prone to spatial bias. We employed multiple approaches to control for spatial bias. We found that (a) functional connectivity is reduced in the SOZ region and accurately lateralizes the SOZ, (b) a model using only spatial sampling, ignorant of intracranial EEG data, achieves moderate performance in localizing the SOZ electrodes, and (c) a model incorporating interictal EEG biomarkers outperforms a model using spatial sampling information alone. Constructing a spatial null model is a useful method to assess how well we can predict seizure generators prior to ever examining intracranial EEG data, and can serve as a benchmark against which to compare performance of machine learning algorithms using intracranial EEG data.

## Data Availability

The data that support the findings of this study are openly available at the following URL/DOI: www.ieeg.org.
